# Changes in tear film osmolarity after 25G+ PPV

**DOI:** 10.1186/s12886-020-01722-4

**Published:** 2020-11-18

**Authors:** J. Němčanský, A. Kopecký, P. Mašek

**Affiliations:** 1 Ophthalmology Clinic, University Hospital Ostrava, 17. Listopadu 1790, 708 52 Ostrava-Poruba, Czech Republic; 2 Department of Craniofacial Surgery, Faculty of Medicine, University of Ostrava, Syllabova 19, 703 00 Ostrava–Vítkovice, Czech Republic

**Keywords:** Pars plana vitrectomy, Anterior segment, Osmolarity, Ocular surface disease, Tear film

## Abstract

**Background:**

The aim of our study was to assess changes of tear film osmolarity after micro-incision 25G+ pars plana vitrectomy (PPV) in a prospective study.

**Methods:**

The group consisted of 21 patients (17 women, 4 men) with an average age of 70,52 years [48; 85]. All patients underwent 25G + PPV surgery due to a disorder of the vitreomacular interface (macular hole or epimacular membrane). Only patients who did not use artificial tears before the surgery and who had not been diagnosed with dry eye syndrome at ours or another institution were included in the study. Except cataract surgery, all ocular surface diseases, intraocular diseases, trauma or surgery were exclusion criterias.

Tear film osmolarity was measured in both eyes in every patient before surgery, 10 days after surgery and 30 days after surgery. A paired test was used for statistical evaluation.

**Results:**

No statistically significant change in osmolarity was found in the operated eyes (*p* > 0.05). No statistically significant changes in time (*p* > 0.05) were found when both eyes were compared. There were no postoperative complications or failure to observe the study protocol.

**Conclusion:**

Micro-incision 25G + PPV does not affect the osmolarity of the tear film.

## Search strategy and selection criteria

For the discussion and review, we have searched PubMed and Web of Science databases for scientific articles on the presented topic. As there is a relative dearth of literature, we used as many published articles with related terms as possible. Some articles were based on a very small samples.

## Background

The tear film protects the eye surface and takes part in refraction. Traditionally, it is divided into 3 layers - aqueous, lipid and mucin [1]. Recently, however, there is more talk about the muco-aqueous, a single layer forming a compact gel [1]. Tear film dysfunction is called dry eye, but the term ocular surface disease is common.

Ocular surface or dry eye disease is a multi-factorial disorder of the ocular surface due to loss of balance of the tear film, and ocular symptoms, in which tear film instability and hyperosmolarity, ocular surface inflammation and damage, and neurosensory abnormalities play etiological roles [2].

The above implies that the tear film’s insufficiency or instability manifests as hyperosmolarity [3]. Evaluation of tear film osmolarity is therefore an important examination in the diagnosis of ocular surface disease. However, it should be noted that the tear film hyperosmolarity can also develop in patients with normal measured values. In addition to this, osmolarity does not correlate very well with symptoms and objective findings [4].

Tear film osmolarity reflects the balance between tear production, evaporation, dissipation and absorption [5]. There are several articles about changes of tear film osmolarity after various anterior segment surgeries. Our aim was to find out whether these changes in the tear film are possible after 25G+ PPV. Even though this type of surgery is without any doubts for the posterior segment of the eye, the ports are introduced via the conjunctiva and there is often at least mild reaction of the conjunctiva after the surgery. Therefore we would like to find out whether the 25G+ PPV can induce any changes in tear film osmolarity.

### Tearlab®

TearLab® osmolarity measurements are considered accurate and, according to some publications, a very objective assessment of the severity of the dry eye [6–9]. Unlike other osmolarity measurement methods, TearLab® is easy to perform in everyday clinical practice.

It is an osmometer that only needs around 50 nl tears to measure the tear film osmolarity. The measurement itself is based on electrical impedance [10]. The examination is very quick, easy for the patient and staff and easy to repeat. It is important not to affect the examination by other factors (dripping drops, other eye examinations). For this reason, it is usually included at the beginning of the examination.

## Methods

The aim of the study was to evaluate changes in tear film osmolarity after 25G + PPV in a narrowly selected group of patients. The results were then compared with the available literature on changes in osmolarity after intraocular operations.

In this study, patients underwent 25G + PPV. All operations were performed in a standardized manner such as sutureless surgery, with minimal cryoretinopexy and using a gas filling.

25G PPV is considered to be a safe microincision technique used on the anterior segment of the eye for the abovementioned reasons. Possible eye changes following the operation (e.g. intraocular pressure, corneal thickness or other anterior segment changes) are reported as temporary [11]. Osmolarity examinations were carried out with the TearLab® instrument on both eyes always before surgery (on the day of the surgery or the preceding day), then after 10 days (± 2 days) and after 30 days (± 3 days). The osmolarity was always measured as the first examination in the morning (7–9 A.M.) after arrival at the clinic. Only after that the next examination (eg visus, intraocular pressure ...) were performed, patients were instructed not to apply any drops at least 2 h before the examination.

TearLab® examination was performed in every patient in the same manner. Original TearLab® cartridges were used, one cartridge for one measurement on one eye. Tear film sample was collected from the tear film fluid near eyelid margin as recommended by the manufacturer of the device. When error was noticed or the sample was not readable, the measurement was always immediately repeated with new original cartridge.

All patients were given the same drops during the follow-ups. On day 1, all received *Dexamethasonum* 1 mg/ml and *Levofloxacinum hemihydricum 5 mg/ml* eye drops by default. Both drops were discontinued according to instructions 7 days before the last follow-up. During the study period, patients were instructed not to apply any artificial tears.

The results were statistically evaluated and then interpreted. A paired test was used for statistical evaluation. For comparison with the available literature, we went through the PubMED and Web of Science databases. The searched terms were: tear osmolarity, tearfilm osmolarity, tear osmolarity surgery, tearfilm osmolarity surgery, intraocular surgery osmolarity and PPV osmolarity. We went through about 600 records in total, but there was not much literature on the relationship between osmolarity and intraocular surgery in indexed journals. All relevant articles were used in the discussion at the end of the article. We analyzed the available articles and used important data from them for our discussion.

The group consisted of 21 patients (17 women, 4 men) with an average age of 70,52 years [48–85 years, SD 78884]. All patients underwent 25G + PPV surgery due to a disorder of the vitreomacular interface (macular hole or epimacular membrane).

Only patients who did not use artificial tears before surgery and who had not been diagnosed with dry eye syndrome at ours or another institution were included in the study. Exclusion criteria were regular active application of any eye drops (artificial tears, antiglaucomatics, etc.), eye surgery in the past (with the exception of uncomplicated cataract surgery, at least 30 days of active eye surface disease (eg. pinguecula, pterygium, conjunctivitis), an obvious low patient compliance and any other eye disease that the investigator believed could affect the results.

Only patients who underwent cataract surgery long time before enrollment to the study were included in the study. No patients after cataract surgery who had complications during the surgery or afterwards, or who used any chronic medication because of the surgery were included in the study.

All patients enrolled in the study were indicated for uncomplicated pars plana vitrectomy (PPV) with a diagnosis of the macular hole or epiretinal membrane. Patients undergoing PPV due to other diagnosis were not included.

None of the patients enrolled were a contact lens wearer.

## Results

The results are presented in (Tables 1, 2).

**Table 1:** Table shows the measured results before the surgery, 10 days after surgery and 30 days after surgery

	Before surgery		10 Days after surgery		30 days after surgery	
	Avg	SD	Avg	SD	Avg	SD
Operated Eye (mOsmol / L)	299.00	11.549	303.86	18.180	297.38	16.375
Heatlhy Eyes (mOsmol / L)	303.33	15.389	301.90	13.160	299.10	13.946

**Table 2:** Shows all measurements of osmolarity before the surgery (V0), 10 days after surgery (V1) and 30 days after surgery (V2). OD is for right eye, OS for left. The measurements of the eye with surgery is always in the first column in the table

Age	Eye with surgery	V0		V1		V2	
		Surgery	Fellow Eye	Surgery	Fellow Eye	Surgery	Fellow Eye
85	OD	311	304	287	286	303	313
66	OD	321	317	308	299	290	302
48	OD	295	287	288	291	287	299
57	OD	324	301	303	322	305	313
75	OD	289	295	313	297	309	295
70	OD	295	294	302	298	306	293
73	OS	300	333	297	330	290	320
67	OS	300	312	300	307	299	308
79	OS	308	324	304	320	326	332
71	OD	296	291	292	297	284	294
65	OD	308	304	298	304	279	304
82	OS	313	332	292	307	290	308
75	OD	282	283	310	299	279	276
73	OD	299	298	355	326	302	302
72	OS	292	296	297	288	286	277
68	OS	292	289	299	286	289	280
65	OD	292	327	354	301	349	301
77	OD	293	294	295	290	289	296
72	OS	280	283	289	308	285	290
71	OS	290	302	297	288	298	292
80	OS	299	304	301	296	300	286

The mean osmolarity of the operated eyes before surgery was 299.00 mOsmol / L [280; 324], (SD 11.549), the mean osmolarity of the non-operated eyes was 303.33 mOsmol / L [283, 33] (SD 15.389).

The mean osmolarity of the operated eyes 10 days after surgery was 303.86 mOsmol / L [289; 335] (SD 18.180), the mean osmolarity of the non-operated eyes was 301.90 mOsmol / L [286; 330] (SD 13.160).

The mean osmolarity of the operated eyes 1 month after the operation was 297.38 mOsmol / L [285; 349] (SD 16.375), the mean osmolarity of the non-operated eyes was 299.10 mOsmol / L [276; 332] (SD 13.946).

No statistically significant change in osmolarity was found in the operated eyes (*p* > 0.05). The preoperative vs post-operative changes over time were compared. A paired test was used for statistical evaluation.

**Fig. 1:**
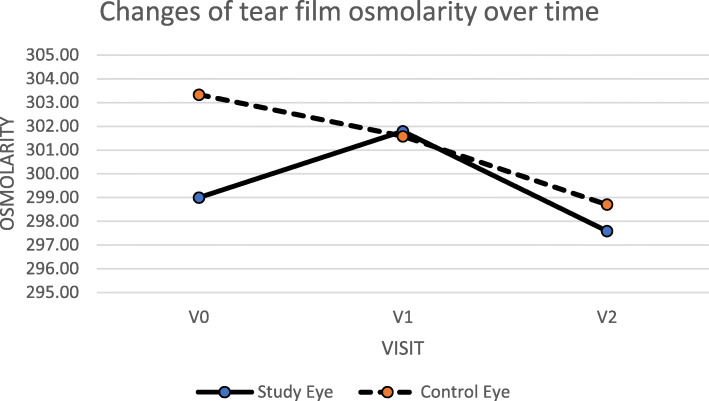
The graph shows the distribution of tear film osmolarity in eyes in that the PPV was performed and in control eyes over the time. There was no statistically significant difference between these two groups (paired sample test used, *p* = 0,644)

No statistically significant changes in time (*p* > 0.05) were found when both eyes were compared. No statistically significant change in tear osmolarity in the control eyes was observed between each time point. Figure 1 shows pre- and post-operative changes of tear osmolarity in control eyes vs cases.

There were no postoperative complications or failure to observe the study protocol.

## Discussion

We found no published studies on changes in osmolarity after PPV. In general, there is a dearth of information on changes in osmolarity after ophthalmological operations. Although it is commonly believed that PPV does not affect the quality of the eye surface, it has been shown that PPV causes some changes on the surface of the eyeball - inter alia, an increase in interleukin concentrations [12]. In general, surgery can be considered a form of trauma, which naturally leads to an inflammatory reaction [13]. This suggests that changes to the eye surface may occur after PPV, although we know that PPV does not affect the anterior segment in the long term [11]. However, we know from clinical practice that even uncomplicated PPV causes, for example, chemosis and conjunctival hyperaemia. These factors may lead to the assumption that osmolarity may change after PPV.

There are reports that evaluate changes in osmolarity after cataract surgery. The conclusions of these studies vary. Some describe a short-term increase in osmolarity after cataract surgery [14]. However, others show that after cataract surgery there is no statistically significant change in tear film osmolarity [15, 16]. Similarly, an increase in osmolarity was confirmed in patients with trabeculectomy. However, Lee et al. did not take into account pre-existing dry eye syndrome or ophthalmological medications [17].

Pterygium surgery is not an intraocular operation, but given that the surgeon works on a bulbar conjunctiva, among other things in the anatomical area of ​​the conjunctiva above the pars plana that is affected by PPV, the data may be relevant. In general, pterygium alone has been shown to increase tear film osmolarity [18]. It is not surprising, therefore, that pterygium surgery, as one of the few ophthalmic operations, reduces tear film osmolarity in the long run. This study also suggested osmolarity binding to pterygium itself - pterygium recurrence causes increased osmolarity [19].

However, it is clear from our results that no statistically significant changes in osmolarity were observed. In general, the prevailing opinion is that inflammation or damage to the eye surface (which can be considered due to surgery) leads to hyperosmolarity, but this was not confirmed on our sample of patients.

One drawback of our study is relatively small small size albeit we took precautions to create samples for achieving the most objectivity. For example, some published papers take no account of drops applied to the eye during the postoperative period or drops applied prior to surgery. In addition, all patients underwent both technically similar operations, which increases the objectivity of our results.

## Conclusion

Our study appears to be among the first to investigate changes in osmolarity after micro-incision vitrectomy. Micro-incision uncomplicated 25G + PPV does not affect the osmolarity of the tear film in patients who did not suffer from other eye or systemic diseases. The question is whether PPV performed in complicated diagnoses such as diabetic retinopathy, emotions, or trauma can affect tear film osmolarity, therefore it is desirable that further research be undertaken to investigate osmolarity in both more complicated PPV and patients with associated diagnoses.

## Data Availability

All data are provided in the article. The datasets used and/or analysed during the current study available from the corresponding author on reasonable request.

## Abbreviation

PPV: Pars plana vitrectomy

## References

[1] Cher I (2008). A new look at lubrication of the ocular surface: fluid mechanics behind the blinking eyelids. Ocul Surf.

[2] Craig JP, Nichols KK, Akpek EK, Caffery B, Dua HS, Joo CK, Liu Z, Nelson JD, Nichols JJ, Tsubota K, Stapleton F (2017). TFOS DEWS II definition and classification report. Ocul Surf.

[3] Thulasi P, Djalilian AR (2017). Update in current diagnostics and therapeutics of dry eye disease. Ophthalmology.

[4] Amparo F, Jin Y, Hamrah P, Schaumberg DA, Dana R (2014). What is the value of incorporating tear osmolarity measurement in assessing patient response to therapy in dry eye disease?. Am J Ophthalmol.

[5] Tomlinson A, Khanal S (2005). Assessment of tear film dynamics: quantification approach. Ocul Surf.

[6] Yoon D, Gadaria-Rathod N, Oh C, Asbell PA (2014). Precision and accuracy of TearLab osmometer in measuring osmolarity of salt solutions. Curr Eye Res.

[7] Masmali A, Alrabiah S, Alharbi A, El-Hiti GA, Almubrad T (2014). Investigation of tear osmolarity using the TearLab Osmolarity system in normal adults in Saudi Arabia. Eye Contact Lens.

[8] Benelli U, Nardi M, Posarelli C, Albert TG (2010). Tear osmolarity measurement using the TearLab Osmolarity system in the assessment of dry eye treatment effectiveness. Cont Lens Anterior Eye.

[9] Kim M, Kim HS, Na KS (2017). Correlation between tear osmolarity and other ocular surface parameters in primary Sjögren's syndrome. Korean J Ophthalmol.

[10] Moisseiev E, Kinori M, Moroz I, Priel E, Moisseiev J (2016). 25-gauge Vitrectomy with epiretinal membrane and internal limiting membrane peeling in eyes with very good visual acuity. Curr Eye Res.

[11] Kopecky A, Nemcansky J (2019). Changes in the anterior segment of the eye following uncomplicated pars plana vitrectomy. A review. Biomed Pap Med Fac Univ Palacky Olomouc Czech Repub.

[12] Fujita A, Uchino E, Otsuka H, Arimura N, Noda Y, Ishibashi T, Sakamoto T (2011). Ocular surface molecule after transconjunctival vitrectomy. Br J Ophthalmol.

[13] Wei Y, Asbell PA (2014). The core mechanism of dry eye disease is inflammation. Eye Contact Lens.

[14] Elksnis Ē, Lāce I, Laganovska G, Erts R (2018). Tear osmolarity after cataract surgery. J Curr Ophthalmol.

[15] Venincasa VD, Galor A, Feuer W, Lee DJ, Florez H, Venincasa MJ (2013). Long-term effects of cataract surgery on tear film parameters. ScientificWorldJournal.

[16] González-Mesa A, Moreno-Arrones JP, Ferrari D, Teus MA (2016). Role of tear osmolarity in dry eye symptoms after cataract surgery. Am J Ophthalmol.

[17] Lee SY, Wong TT, Chua J, Boo C, Soh YF, Tong L (2013). Effect of chronic anti-glaucoma medications and trabeculectomy on tear osmolarity. Eye (Lond).

[18] Julio G, Lluch S, Pujol P, Alonso S, Merindano D (2012). Tear osmolarity and ocular changes in pterygium. Cornea.

[19] Türkyılmaz K, Oner V, Sevim MŞ, Kurt A, Sekeryapan B, Durmuş M (2013). Effect of pterygium surgery on tear osmolarity. J Ophthalmol.

